# Effects of High Doses of Selenate, Selenite and Nano-Selenium on Biometrical Characteristics, Yield and Biofortification Levels of *Vicia faba* L. Cultivars

**DOI:** 10.3390/plants12152847

**Published:** 2023-08-01

**Authors:** Anna Sindireva, Nadezhda Golubkina, Helene Bezuglova, Mikhail Fedotov, Andrey Alpatov, Erdene Erdenotsogt, Agnieszka Sękara, Otilia Cristina Murariu, Gianluca Caruso

**Affiliations:** 1 Department of Geoecology and Nature Management, Tumen State University, Volodarsky Str. 6, 625003 Tumen, Russia; 2 Federal Scientific Vegetable Center, Selectsionnaya, 14, VNIISSOK, Odintsovo District, 143072 Moscow, Russia; 3 Department of Agronomy, Selection and Seeds Production, Omsk State Agrarian University, Institutskaya Square, 1, 644008 Omsk, Russia; ev.bezuglova@omgau.org; 4 A. Baikov Institute of Metallurgy and Material Science, Leninsky Pr., 49, 119334 Moscow, Russia; mikle_fed@mail.ru (M.F.); aaalpatov@imet.ac.ru (A.A.); 5 Mongolian National Center of Public Health, Peace Ave, 46, Ulaanbaatar 211049, Mongolia; erd625@yahoo.com; 6 Department of Horticulture, Faculty of Biotechnology and Horticulture, University of Agriculture, 31-120 Krakow, Poland; agnieszka.sekara@urk.edu.pl; 7 Department of Food Technology, “Ion Ionescu de la Brad” Iasi University of Life Sciences, 3 M. Sadoveanu Alley, 700440 Iasi, Romania; otiliamurariu@uaiasi.ro; 8 Department of Agricultural Sciences, University of Naples Federico II, Portici, 80055 Naples, Italy; gcaruso@unina.it

**Keywords:** *Vicia faba* L., nano-Se, selenate, selenite, seed yield, proteins

## Abstract

Faba bean (*Vicia faba* L.) has spread worldwide as an excellent source of proteins. To evaluate the efficiency of Se biofortification, four cultivars of *V. faba* (Belorussian, Russian Black, Hangdown Grünkernig, and Dreifach Weiβe) were foliar treated with 1.27 mM solutions of nano-Se, sodium selenate, and sodium selenite. Yield, protein, and Se contents were greatly affected by genetic factors and chemical form of Se. Selenium biofortification levels were negatively correlated with Se concentration in control plants and increased according to the following sequence: nano-Se < sodium selenite < sodium selenate. Contrary to selenate and selenite, nano-Se showed a growth-stimulating effect, improving yield, seed weight, and pod number. Pod thickness decreased significantly as a result of nano-Se supply and increased by 1.5–2.3 times under selenate and selenite supply. The highest Se concentrations were recorded in the seeds of Se-fortified cv. Belorussian and the lowest one in those of Se-treated Hangdown Grünkernig. Protein accumulation was varietal dependent and decreased upon 1.27 mM selenate and selenite treatment in the cvs. Hangdown Grünkernig and Dreifach Weiβe. The results indicate the high prospects of nano-Se supply for the production of faba bean seeds with high levels of Se.

## 1. Introduction

The biological activity of selenium (Se), which is essential for humans, relates to its high antioxidant and immunomodulating properties and the ability to substitute sulfur in S-containing organic compounds. Plants actively participate in the mentioned processes, transforming soil inorganic selenates (+6) and selenites (+4) into selenocysteine (SeCys), selenocystine and selenomethionine (SeMet) [1], and other sulfur-containing biologically active compounds (glucosynolates, polyphenols, allicine analogs) [2,3]. To prevent Se toxicity caused by prooxidant properties of Se [4], plants synthetize volatile methylated forms (dimethyl and trimethyl selenides) [5] and methylated forms of amino acids and peptides [2], which are not incorporated into proteins [2]. Taking into account that organic Se forms have high biological activity, beneficial for human health [6], many successful attempts to clarify Se speciation in cereal and legume crops have been made [7,8,9,10]. In this respect, selenate (Se^+6^)-treated wheat accumulated predominantly C-Se-C derivatives (particularly SeMet), while selenite (Se^+4^) supplied to plants led to the formation of C-Se-Se-C compounds, such as selenocystine [8]. As far as legume species are concerned, selenocystein (SeCys) and selenomethionine (SeMet) were detected in Se-fortified soybean [9], while selenomethyl selenocystein (SeMeSeCys) and SeMet were found in *Phraseolus vulgaris* supplemented with sodium selenate [10].

The widespread Se-deficient soils in the world, the importance of Se to protect human organisms against viral, cardiovascular, and oncological diseases, and the genetically determined process of SeCys biosynthesis in humans give rise to the prospects of human Se status optimization. The latter may be carried out via the production of functional food with high levels of Se, aimed at increasing human longevity and decreasing mortality [6]. In this respect, agrochemical Se biofortification of plants, particularly the Fabaceae species, is considered highly beneficial [11] due to the ability of these plants to synthesize biologically active and easily digestible Se-containing proteins, peptides, and amino acids, as well as corresponding methylated forms, characterized by extremely high anti-carcinogenic activity [12].

*Vicia faba* ranks second after soybean in terms of protein accumulation ability, and its cultivation has become particularly widespread in recent years, as a plant with high nutritional value with a high capacity to fix air nitrogen and tolerate environmental stresses [13]. The protein content of faba bean ranges from 24% to 35% of the seed dry matter [14,15], making it a major protein-rich pulse crop [16,17]. According to Rahate et al. [18], faba bean contains almost twice the protein content present in cereal grains, with the predominance of globulins (60%), albumins (20%), glutelins (15%), and prolamins (8%). Among the latter, albumins contain the highest levels of sulfur amino acids, thus being the main target in Se biofortification.

Notably, a lot of information has been gained about the Se biofortification peculiarities of Fabaceae plants. Indeed, faba bean foliar biofortification with sodium selenate revealed a narrow Se concentration range providing a beneficial effect on bean yield and quality [19]. Ravello et al. [20] reported interesting prospects of Fabaceae bean Se biofortification under drought. In 2020, Patel et al. [21] reported a growth-stimulating effect of Se-containing rhizobacteria, resulting in a production of beans with high Se levels. In a pot experiment, Hermosillo-Cereceres et al. [22] recorded higher toxicity of soil supplied with selenite (Se^+4^) than selenate (Se^+6^) to Fabaceae beans. A successful attempt of soybean Se biofortification was achieved using Se-containing phosphorous fertilizer, which increased yield and seed quality [23]. Garden pea biofortification with Se was carried out in a pot experiment [24], whereas foliar application of sodium selenate increased the yield and quality of chickpea [25].

Different approaches of Se biofortification include various methods of Se supply (soil, foliar, or seed soaking) and utilization of different Se chemical forms: selenate (+6), selenite (+4), organic Se derivatives, and Se-nanoparticles (nano-Se) [26]. Regarding selenate use, foliar Se application was the most effective in different agricultural crops [27]. Among Se derivatives, selenates are the most labile forms and selenites the most toxic ones, whereas nano-Se is characterized by low toxicity [2,28]. At high doses, Se toxicity is supposed to be caused by the replacement of S atoms by Se in S-containing amino acids; this results in changes of the structure and activity of Se-substituted proteins and, consequently, in plant growth decrease [29]. Moreover, at over-concentrations, Se acts as a pro-oxidant and catalyzes the oxidation of thiols and simultaneously generates a superoxide that can damage cellular components [30], resulting in metabolic disturbances and yield reduction [31].

There is no information yet about the effect of high nano-Se doses on plant growth and development, but nano-Se application is extremely attractive and relates to its antistressor, growth stimulating, and insecticidal properties [26,32]. Nevertheless, to date, extremely little knowledge is available about nano-Se treatment to pulses. An exception is represented by the work of Gharib et al. [33], who biofortified cowpea by foliar applications of Na_2_SeO_4_ and Se-NPs, which resulted in higher levels of total carbohydrates and proteins. However, using chemically synthesized nano-Se, the mentioned authors did not apply a pure NP, but a mixture of sodium selenate and ascorbic acid. In another research, faba bean seed imbibition in nano-Se solution resulted in yield increase [34].

The lack of detailed data regarding the peculiarities of faba bean biofortification with Se has led to questions about the effects of Se chemical form and genotype on faba bean yield and quality, as well as the degree of plant tolerance to high concentrations of this element. The aim of this research was to evaluate the effects of high doses of nano-Se, selenate, and selenite on yield, biometrical characteristics, and Se and protein accumulation in four broad bean cultivars.

## 2. Results and Discussion

Faba bean does not belong to Se accumulators [2], but it is an important source of proteins and, therefore, entails high prospects of Se biofortification due to the ability of this element to produce Se-enriched amino acids. 

### 2.1. Yield and Biometrical Characteristics

**Figure 1:**
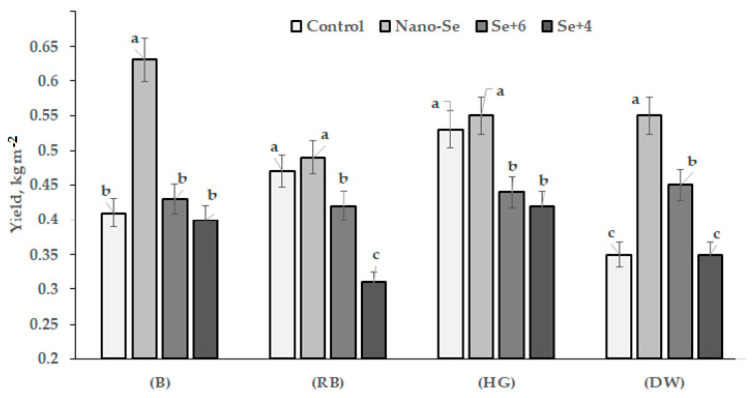
Foliar application effect of nano-Se, sodium selenate, and sodium selenite on faba bean yield. (B) Belorussian; (RB) Russian Black; (HG) Hangdown Grünkernig; (DW) Dreifach Weiβe. Within each cultivar, values with the same letters do not differ statistically according to Duncan test at *p* < 0.05.

**Figure 2:**
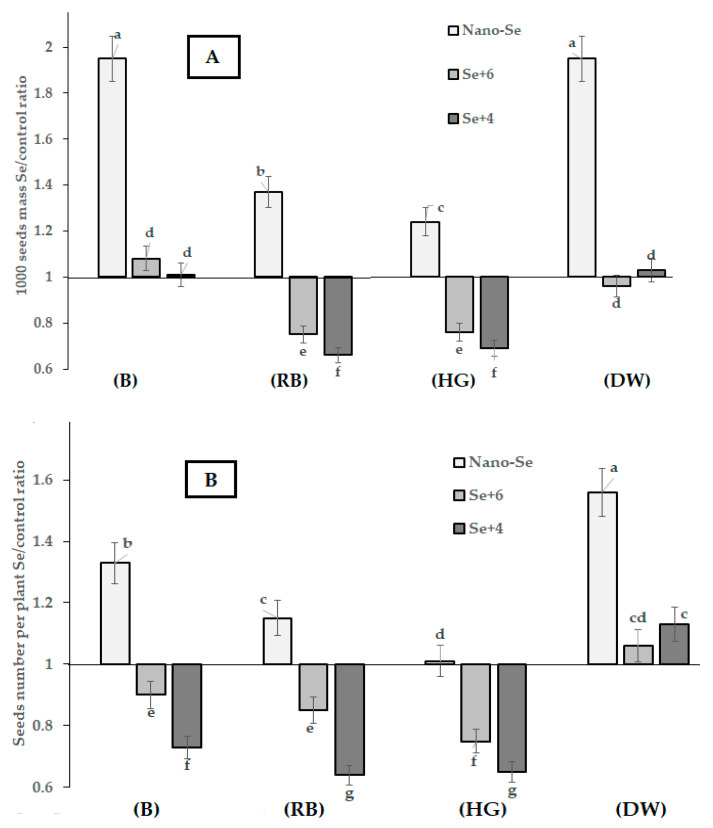

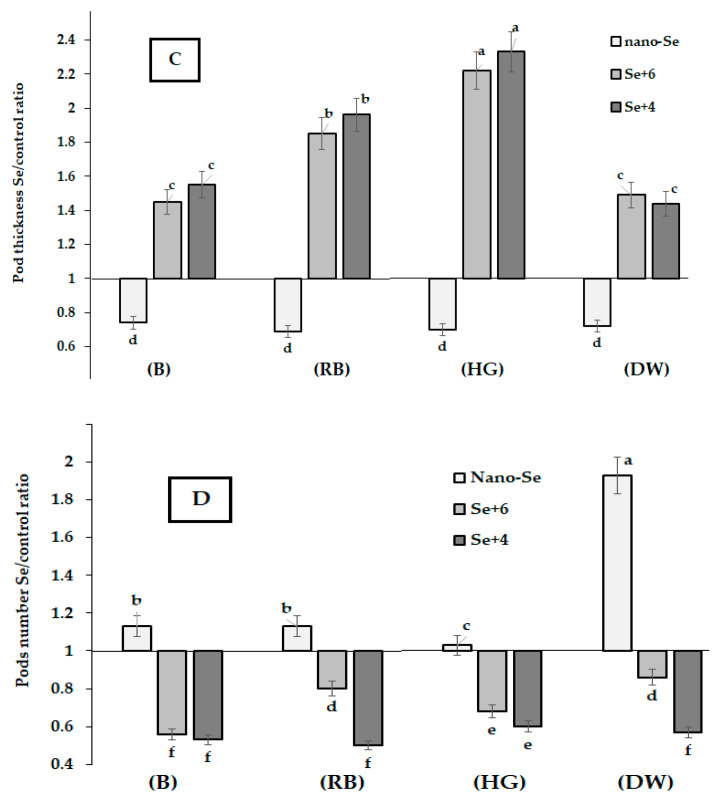
Effect of Se biofortification on 1000 seed weight (**A**), number of seeds per plant (**B**), pod thickness (**C**), number of pods (**D**) of *Vicia faba*. (B) Belorussian; (RB) Russian Black; (HG) Hangdown Grünkernig; (DW) Dreifach Weiβe. Values with the same letters do not differ statistically according to Duncan test at *p* < 0.05.

The results, presented in Table 1, Table 2, Table 3 and Table 4 and in Figure 1 and Figure 2, indicate significant effects of both the genetic variability and the chemical form of Se on plant biometrical parameters, seed yield, weight and number, and pod size. The application of a high Se dose (1.27 mM) gave rise to the first results regarding faba bean varietal reaction to Se toxicity. Indeed, in these conditions, most of the biometrical parameters measured in plants treated with sodium selenate and selenite were significantly lower than those of control plants. Despite genetic differences, a 3-fold mean decrease in the number of fruiting nodes and pods per plant was recorded in plants treated with sodium selenate and selenite. Seed weight and number per plant decreased in these conditions by 10–40%, while the weight of 1000 seeds decreased significantly in the cvs. Russian Black and Hangdown Grünkernig (Table 1, Table 2, Table 3 and Table 4; Figure 1 and Figure 2).

Out of the four cultivars tested, Hangdown Grünkernig showed the highest seed weight of 259 g per 1000 seeds, which was 1.4–1.6 higher than the seed weight of the other cultivars. Faba bean cultivars of Russian selection (Belorussian, Russian Black) were characterized by a 1.5 times higher number of fruiting nodes compared to Dutch cultivars (Hangdown Grünkernig and Dreifach Weiβe). Overall, the number of pods per plant (2.5–10) was significantly lower than those reported in the literature (10–22) [35], which may reflect the higher plant density effect in the present investigation.

The toxic effect of sodium selenate and selenite resulted in a significant decrease in pod number and fruiting nodes, while seed yield did not change (cvs. Belorussian and Dreifach Weiβe) or decreased by 1.12–1.52 and 1.20–1.26 times in cvs. Russian Black and Hangdown Grünkernig, respectively, with the most significant dramatic effect recorded under selenite supply. The results of the present research are consistent with the higher toxicity of selenite (Se^+4^), compared to selenate (Se^+6^), previously recorded by Hermosillo-Cereceres et al. [22] in a pot experiment with Fabaceae beans. The data presented in Figure 1 show high varietal differences of plant tolerance to high Se salt doses, with the most detrimental effect of sodium selenite on Russian Black cultivar.

In contrast, nano-Se significantly stimulated the growth of faba bean (Table 1, Table 2, Table 3 and Table 4; Figure 1), especially cvs. Belorussian and Dreifach Weiβe. In this respect, the largest varietal differences were shown by the two cultivars Belorussian and Russian Black, with seed yields of 0.63 and 0.49 kg m^−2^, respectively (Figure 1). The data presented in Figure 1 indicate that, depending on the cultivar, high selenate doses may affect seed yield, with a significant increase (cv. Dreifach Weiβe) or decrease (cvs. Russian Black and Hangdown Grünkernig), or have no effect (cv. Belorussian). Selenite treatment did not change the seed yield of cvs. Belorussian and Dreifach Weiβe, but significantly decreased the seed yield of cultivars Russian Black and Hangdown Grünkernig. Overall, the highest beneficial effect of nano-Se application was recorded in cvs. Belorussian and Dreifach Weiβe, which entails great prospects of nano-Se utilization for the biofortification of these cultivars. The high efficiency of selenate application was recorded only for cv. Dreifach Weiβe, indicating the high tolerance of this cultivar to high levels of selenate. In contrast, selenite supply did not show beneficial effects in all cultivars tested. Nevertheless, the latter results revealed the highest tolerance of cv. Belorussian to sodium selenite supply (Figure 1). 

From the comparison between the control and Se-treated plants of faba bean in terms of biometrical parameters, both the effect of genotype and of the Se chemical form on seed weight/number and pod thickness/number arose (Figure 2). In this respect, the increase in seed weight due to Se supply was highly significant under nano-Se application for all cultivars studied, whereas no effect or even an inhibition were recorded upon sodium selenate and selenite applications (Figure 2A). The number of pods and seeds were also the highest under nano-Se supply, contrary to the growth inhibition effect of inorganic Se salts (Figure 2B,D). The beneficial effect of nano selenium (nano-Se) in the present work may relate to the well-known role of nano-Se in plants, as a nano-fertilizer, anti-stress, and biostimulant [36]. To date, nano-Se utilization on legume crops has been performed only in cowpea by Li et al. [37], who demonstrated lower toxicity of Se nanoparticles compared to selenite.

The Se supplementation effect on the different faba bean parameters examined revealed that Se nanoparticles inhibited pod thickness, contrary to the effect of sodium selenate and selenite (Figure 2C), thus suggesting a high dependence of the mentioned parameter on plant genetic peculiarities and the Se chemical form (Figure 2C). Taking into account the effects of the growth inhibition of selenate and selenite supply and of the growth stimulation of nano-Se treatment, it can be inferred that pod thickness is supposedly connected with seed protection against Se toxicity, though further investigation is needed to prove this hypothesis.

Seed numbers were also greatly dependent on both Se chemical form and cultivar. In the case of nano-Se, the highest beneficial effect was recorded in cv. Dreifach Weiβe, whereas no effects were recorded in Hangdown Grünkernig under nano-Se supply. The decrease in seed number was the most significant under foliar selenite application, and especially for cvs. Russian Black and Hangdown Grünkernig (Figure 2B).

### 2.2. Se Accumulation

In conditions of high Se doses, the accumulation of this element in faba bean decreased according to the sequence: selenate > selenite > nano-Se (Table 5), while in a spinach investigation [38], Se content was highest under selenate treatment, followed by nano-Se and selenite, with significant differences between male and female forms. The highest differences in biofortification levels between male and female forms of spinach is supposedly due to the participation of phytohormones in Se nanoparticles accumulation. Indeed, plant tolerance to Se nanoparticles is known to be closely connected with phytohormones, such as jasmonic, salicylic acid, and ethylene [7,8]. Kolbert et al. [4] postulated the existence of hormonal disturbances in conditions of Se toxicity. Other authors mentioned the involvement of aquaporins in nano-Se assimilation by plants [39]. The assimilation of selenate via sulphate transporters and of selenite via phosphate transport channels forms the basis of physiological differences in selenate, selenite, and nano-Se assimilation [32]. The results of Se biofortification of cowpea, achieved by Li et al. [37], demonstrated that Se dose plays a pivotal role in producing different biofortification values: at low doses, nano-Se showed higher biofortification levels compared to sodium selenite, while at toxic concentrations, the opposite phenomenon took place.

The data presented in Table 5 also indicate the low ability of cv. Dreifach Weiβe to accumulate all forms of Se tested. Notably, this cultivar is characterized by the lowest pod number per plant and the highest pod length.

**Figure 3:**
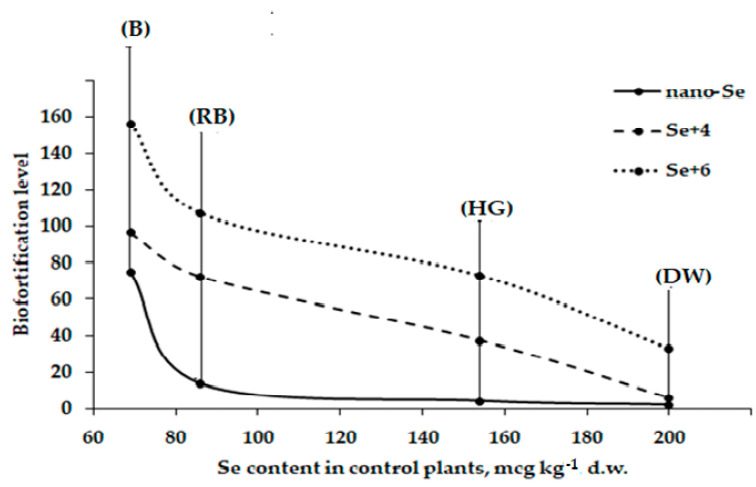
Effect of Se chemical form on biofortification levels of four faba bean cultivars: (B) Belorussian; (RB) Russian Black; (HG) Hangdown Grünkernig; (DW) Dreifach Weiβe.

The present data also indicate that biofortification level is closely related to the seed Se concentration of control plants. The data trends in Figure 3 show that the biofortification level of faba bean increases significantly with the decrease of Se content in control plants. Indeed, the ability to accumulate Se greatly differs between cultivars Belorussian, Russian Black, Hangdown Grünkernig, and Dreifach Weiβe. 

The comparison of seed Se levels in the *Vicia faba* cultivars examined, based on the daily Se consumption requirement (70 µg·day^−1^), reveals that 50 g seed consumption may provide from 30% to 86% adequate consumption level (ACL) from nano-Se-treated plants, 85% to 474% from the sodium-selenite-supplied ones, and 470% to 800% from the selenate-treated faba bean. In this respect, *Vicia faba* treated with 10 mg·L^−1^ nano-Se is the most suitable for functional food production. Growth inhibition of *V. faba* supplied with high concentrations of selenite and selenate and the extremely high Se consumption level deriving from Se^+4^ and Se^+6^-treated seeds provide prospects of lower Se dose utilization to improve yield and quality, thus obtaining a product with optimal Se content.

### 2.3. Protein

**Figure 4:**
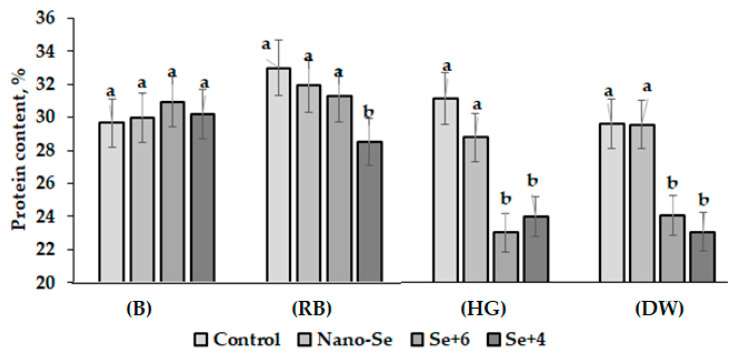
Protein content in faba bean cultivars as affected by Se supply: (B) Belorussian; (RB) Russian Black; (HG) Hangdown Grünkernig; and (DW) Dreifach Weiβe. Within each cultivar, values with the same letters do not differ statistically according to Duncan test at *p* < 0.05.

Selenium is known to affect amino acid biosynthesis in plants [40]. The biofortification of soybean with Se promoted protein synthesis with the predominance of SeCys and SeMet formation [9]. In this respect, the beneficial effect of Se biofortification is considered one of the most important impacts of Se supply. Nevertheless, the present results indicate that high Se concentrations produce unfavorable conditions for protein accumulation in faba bean. Indeed, the chosen Se concentration (1.27 mM) did not stimulate protein accumulation either in the form of nano-Se or sodium selenate and sodium selenite in faba bean, but protein accumulation even significantly decreased as a result of selenite or selenate application to cvs. Hangdown Grünkernig and Dreifach Weiβe (Figure 4). 

## 3. Material and Methods

### 3.1. Growing Conditions and Experimental Protocol

Research was conducted on faba bean (*Vicia faba* L.) in 2019–2021 from April to October at the experimental fields of Omsk State University, Russia (54°58′ N, 73°23′ E), in a meadow-chernozem, thin, heavy loamy soil with the following characteristics: pH 6.8; 5.2–6.5% organic matter, 19 mg kg^−1^ mineral nitrogen; 60 mg kg^−1^ d.w. mobile phosphorous; 90 mg kg^−1^ d.w. exchangeable potassium; 19.8–23.3 mg-eq 100 g^−1^ d.w. Ca; 326 ± 91 µg kg^−1^ d.w. Se.

Mean values of monthly temperature and precipitation during the crop cycles are presented in Table 6.

The experimental protocol was based on the comparison between three Se treatments plus a non-treated control, within four faba bean cultivars of Russian (Belorussian, Rassian Black) and Dutch selection (Dreifach Weiβe and Hangdown Grünkernig), using a randomized complete block design with three replicates. Seeds of Russian cultivars were from the Federal Scientific Vegetable Center, while seeds of Dutch cultivars were obtained from the Magic Garden Seeds production EU. The experimental unit had a 2 m^2^ surface area including 20 plants. The following Se treatments were applied: (1) non-treated control; (2) foliar supply of sodium selenite (220 mg L^−1^); (3) foliar supply of sodium selenate (240 mg L^−1^); and (4) foliar supply of Se nanoparticles (100 mg L^−1^). The concentration of Se was 1.27 mM in all treatments. Single spraying was carried out at the beginning of the flowering phase (8–10 July) at a dose of 1 L·m^−2^.

Crop harvesting was performed on 12 September.

After harvesting, the following variables were measured: yield, seed weight and number, stem length, pod width, nodes number up to the first pod, number of fruiting nodes, pod width, thickness and number per plant, and number of branches. To evaluate the effect of Se on biometrical parameters, the ratios between the values of Se-treated and control plants were calculated and compared.

All the faba bean seeds harvested in each plot were milled to a fine powder and used for the determination of Se and protein content.

### 3.2. Preparation and Characterization of Selenium Colloidal Solution

Nanoparticles of Se were acquired using pulse laser ablation in deionized water. A solid gray Se target was placed at the base of a stationary glass container filled with deionized water. The target was subjected to nanosecond Nd:YAG laser irradiation with a wavelength of 1064 nm, pulse duration of 12 ns, and energy of 2.5 J in pulse, which was concentrated on the target using a lens.

The Se nanoparticle concentration was analyzed via inductively coupled plasma atomic emission spectrometry (ICP-AES) using an ULTIMA 2 (Horiba Jobin Yvon, Palaiseau, France) spectrometer. 

**Figure 5:**
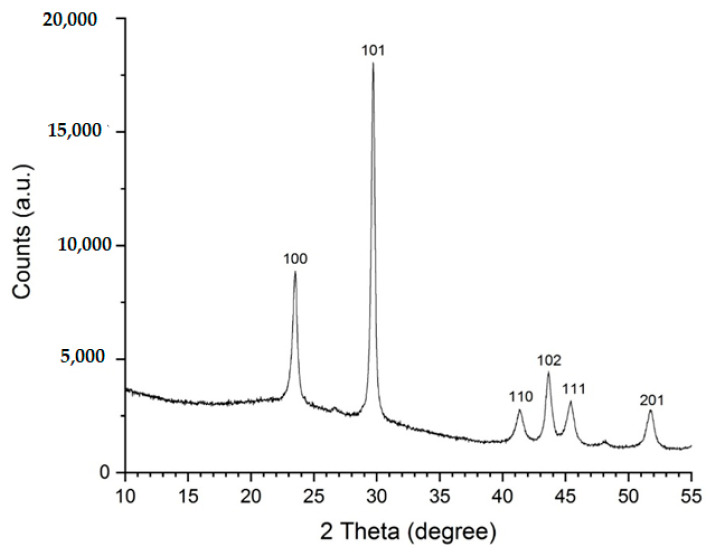
X-ray diffraction pattern of Se nanoparticles.

The phase characterization of Se nanoparticles was analyzed via X-ray diffraction (XRD) using an X-ray diffractometer «Shimadzu XRD-600» (Figure 5). The diffraction patterns were studied in the 2θ range from 10° to 55° at a tube voltage of 40 kV and a current of 100 mA. The diffraction peaks were determined via a comparison with literature data and use of the ICDD database (International Centre for Diffraction Data Power Diffraction File; 2 Campus Blvd: Newtown Square, PA, USA, 2007).

**Figure 6:**
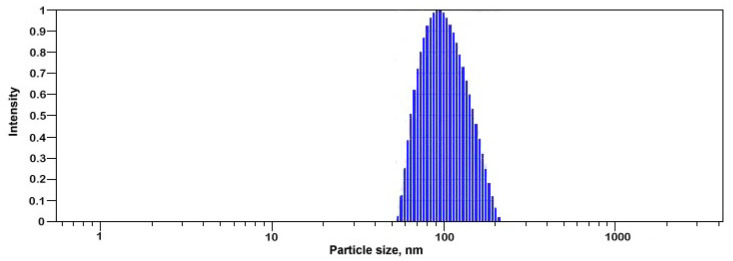
Size distribution of Se nanoparticles in colloidal solution.

To obtain Se nanoparticles, the colloidal solution was dried at 50 °C. The resulting precipitate was examined using XRD (Figure 6). Selenium nanoparticles showed characteristic diffraction peaks at 2θ values of 23.7, 29.8, 41.3, 43.7, 45.5, and 51.7, corresponding to crystal planes of crystalline Se (100, 101, 110, 102, 111, and 201).

Size distribution and zeta potential of Se nanoparticles were analyzed via Dynamic Light Scattering (DLS) using a Photocor Compact Z (Photocor, Beltsville, MD, USA) laser analyzer with a wavelength λ = 589 nm and laser rated-power output of 32 mW at 25 °C.

DLS showed that the Se nanoparticles had a narrow size distribution and an average size of 90 nm (Figure 6). The ζ-potential of the Se colloidal solution was −36.2 mV, suggesting that the Se nanoparticles in colloidal solution tended to repel each other against aggregation.

### 3.3. Selenium

Selenium was analyzed using the fluorometric method previously described for tissues and biological fluids [41]. Dried homogenized samples were digested via heating with a mixture of nitric–perchloric acids, subsequent reduction of selenate (Se^+6^) to selenite (Se^+4^) with a solution of 6 N HCl, and the formation of a complex between Se^+4^ and 2,3-diaminonaphtalene. Selenium concentration was calculated by recording the piazoselenol fluorescence value in hexane at 519 nm λ emission and 376 nm λ excitation. Each determination was performed in triplicate. The precision of the results was verified using a reference standard-lyophilized mitsuba stem in each determination with a Se concentration of 1865 µg·kg^−1^ (Federal Scientific Vegetable Center). The results are expressed in µg·kg^−1^ d.w. 

### 3.4. Protein Content

The crude protein content was measured using the Kjeldahl methodology, based on sample digestion with sulfuric acid, and quantification of the ammonia after the reaction mixture alkalization [42]. 

### 3.5. Statistical Analysis

Data were processed using analysis of variance, and mean separations were performed through the Duncan’s multiple range test, with reference to 0.05 probability level, using SPSS software version 27 (Armonk, NY, USA).

## 4. Conclusions

The present investigation has demonstrated high faba bean varietal differences in Se accumulation under nano-Se, sodium selenate, and sodium selenite supply. The plants showed the highest tolerance to nano-Se, and the lowest tolerance to selenite treatment. Compared to Russian cultivars (Belorussian, Russian Black), the Dutch varieties (Hangdown Grünkernig and Dreifach Weiβe) were characterized by lower protein content and nano-Se accumulation, but significantly higher Se content in seeds of control plants. Contrary to nano-Se-treated plants, the plants fortified with selenate and selenite had higher thickness of pods, which were fewer in number. Among the four cultivars tested, cv. Belorussian showed the highest yield, seed weight and quality, biofortification levels, and tolerance to high Se concentration. The revealed peculiarities of Se biofortification of faba bean plants provide high prospects of nano-Se utilization for functional food production.

## Figures and Tables

**Table 1:** Biometrical parameters of faba bean, cv. Belorussian, under different Se form supply.

Parameter	Control	Nano-Se (Se°)	Selenate (Se^+6^)	Selenite (Se^+4^)
Stem length (cm)	81.3 b	85.6 a	81.4 b	80.5 b
Nodes number up to the first pod	3.5 b	4.4 a	2.6 c	2.5 c
Number of fruiting nodes	7.5 a	8.5 a	3.6 b	3.4 b
Pod number per plant	7.5 a	8.5 a	4.2 b	4.0 b
Pod length (cm)	9.0 b	9.6 ab	10.4 ab	11.0 a
Pod width (cm)	2.0 a	2.0 a	1.8 a	2.0 a
Pod thickness (cm)	3.8 b	2.8 c	5.5 a	5.9 a
Seed number per pod	3.2 a	3.6 a	3.4 a	3.3 a
Seed number per plant	16.0 b	21.3 a	14.4 b	11.7 c
Seed weight per plant (g)	35.7 a	35.1 a	24.9 b	18.2 c
Weight of 1000 seeds (g)	162.3 b	316.0 a	174.8 b	164.4 b

Along each line, values with the same letters do not differ statistically according to Duncan test at *p* < 0.05.

**Table 2:** Biometrical parameters of faba bean, cv. Russian Black, under different Se form supply.

Parameter	Control	Nano-Se (Se°)	Selenate (Se^+6^)	Selenite (Se^+4^)
Stem length (cm)	71.4 b	81.1 a	77.3 a	73.7 b
Nodes number up to the first pod	2.5 b	3.5 a	3.2 a	2.5 b
Number of fruiting nodes	7.8 c	8.1 b	4.2 a	3.8 d
Pod number per plant	9.0 b	10.2 ab	7.2 a	4.5 c
Pod length (cm)	7.0 a	8.0 a	6.8 a	7.6 a
Pod width (cm)	1.6 b	1.7 ab	1.9 a	1.7 ab
Pod thickness (cm)	2.6 c	1.8 d	4.8 b	5.1 a
Seed number per pod	3.0 a	2.7 a	2.6 a	2.8 a
Seed number per plant	17.7 ab	20.4 a	15.1 b	11.3 c
Seed weight per plant (g)	21.0 b	28.8 a	15.7 c	13.8 c
Weight of 1000 seeds (g)	189.2 b	260.0 a	142.0 c	124.6 c

Along each line, values with the same letters do not differ statistically according to Duncan test at *p* < 0.05.

**Table 3:** Biometrical parameters of faba bean, cv. Hangdown Grünkernig, under different Se form supply.

Parameter	Control	Nano-Se (Se°)	Selenate (Se^+6^)	Selenite (Se^+4^)
Stem length (cm)	80.3 a	80.8 a	76.2 b	72.4 c
Nodes number up to the first pod	3.4 a	3.7 a	3.5 a	2.7 b
Number of fruiting nodes	5.8 a	5.3 a	3.5 b	3.5 b
Pod number per plant	6.0 a	6.2 a	4.1 b	3.6 b
Pod length (cm)	10.8 a	9.6 a	10.8 a	11.6 a
Pod width (cm)	2.0 a	1.9 a	2.2 a	2.0 a
Pod thickness (cm)	2.7 b	1.9 c	6.0 a	6.3 a
Seed number per pod	3.7 ab	4.1 a	3.3 b	3.5 ab
Seed number per plant	16.2 a	16.4 a	12.2 b	10.6 b
Seed weight per plant (g)	28.7 a	25.5 ab	21.7 bc	19.7 c
Weight of 1000 seeds (g)	259.0 b	320.0 a	196.0 c	178.0 c

Along each line, values with the same letters do not differ statistically according to Duncan test at *p* < 0.05.

**Table 4:** Biometrical parameters of faba bean, cv. Dreifach Weiβe, under different Se form supply.

Parameter	Control	Nano-Se (Se°)	Selenate (Se^+6^)	Selenite (Se^+4^)
Stem length (cm)	81.0 b	85.6 a	77.5 bc	73.6 c
Nodes number up to the first pod	2.8 b	4.4 a	2.0 c	2.4 bc
Number of fruiting nodes	4.2 b	8.5 a	2.1 d	3.0 c
Pod number per plant	4.4 b	8.5 a	3.8 b	2.5 c
Pod length (cm)	12.6 a	9.6 b	11.2 ab	13.3 a
Pod width (cm)	2.2 a	2.0 ab	1.8 b	1.9 ab
Pod thickness (cm)	3.9 bc	2.8 c	5.8 a	5.6 a
Seed number per pod	3.2 b	3.6 ab	3.9	4.1 a
Seed number per plant	10.2 b	15.9 a	10.8	11.5 b
Seed weight per plant (g)	17.9 b	35.1 a	17.2 b	20.0 b
Weight of 1000 seeds (g)	162.3 b	316.0 a	155.0 b	167 b

Along each line, values with the same letters do not differ statistically according to Duncan test at *p* < 0.05.

**Table 5:** Selenium content in *Vicia faba* seeds.

Cultivar	Se Content (mg kg^−1^ d.w.)			
	Control	Nano-Se	Sodium Selenite	Sodium Selenate
Russian Black	0.086 ± 0.004 c	1.180 ± 0.046 b	6.231 ± 0.012 a	9.197 ± 0.180 b
Belorussian	0.069 ± 0.004 d	5.172 ± 0.116 a	6.655 ± 0.585 a	10.758 ± 0.032 ab
Hangdown Grünkernig	0.154 ± 0.016 b	0.652 ± 0.084 c	5.754 ± 0.204 a	11.249 ± 1.510 a
Dreifach Weiβe	0.200 ± 0.009 a	0.426 ± 0.020 d	1.195 ± 0.254 b	6.596 ± 0.309 c

Along each line, values with the same letters do not differ statistically according to Duncan test at *p* < 0.05.

**Table 6:** Mean values of monthly temperature and precipitation during the crop cycles in 2019–2021.

Month	2019		2020		2021	
	Temperature (°C)	Precipitation (mm)	Temperature (°C)	Precipitation (mm)	Temperature (°C)	Precipitation (mm)
May	12.2	37.8	17.4	13	17.3	13
June	15.5	85.3	16.1	45	16.9	45
July	20.4	28.9	21.2	33	20.6	33
August	18.0	40.5	19.4	43	19.1	43
September	10.8	48.2	11.4	44	10.7	44

## Data Availability

Not applicable.
